# Decoding the Aroma Gap: Sensomics-Based Characterization of Key Odorants in the Plant-Based Egg and Chicken Egg

**DOI:** 10.1021/acs.jafc.5c04304

**Published:** 2025-05-22

**Authors:** Thi Khanh Linh Tran, Elodie Gillich, Amandine André, Marie-Louise Cezanne, Peter Gläser, Imre Blank, Irene Chetschik

**Affiliations:** † Life Sciences and Facility Management, 111833 Zurich University of Applied Sciences (ZHAW), 8820 Wädenswil, Switzerland; ‡ Lovely Day Foods GmbH, Schönhauser Allee 176, 10119 Berlin, Germany

**Keywords:** chicken and vegan egg, dose over threshold factors, aroma extract dilution analysis, stable isotope dilution assay, aroma reconstitution

## Abstract

This study presents the first molecular-level comparative analysis of chicken scrambled eggs and vegan scrambled eggs, identifying a total of 20 odorants using Aroma Extract Dilution Analysis (AEDA). Quantitation via Stable Isotope Dilution Assay (SIDA) and calculation of dose over threshold (DoT) factors revealed 15 key odorants with DoT factors > 1 in scrambled chicken eggs, while scrambled vegan eggs exhibited 11 key odorants with DoT factors > 1. Notably, the vegan product lacked five critical compounds: (*E,Z*)-2,6-nonadienal (cucumber-like), 2,3-butanedione (butter-like), (*E*)-2-nonenal (fatty), 4-methylphenol (smoky), and 2,3-diethyl-5-methylpyrazine (earthy), while it displayed a higher odor activity for 1-octen-3-one (mushroom-like), hexanal (grassy), 3-methylbutanoic acid (sweaty), and *trans*-4,5-epoxy-(*E*)-2-decenal (metallic-like). The aroma reconstitution experiment confirmed that key odorants of scrambled chicken egg aroma were successfully decoded. Omission experiments further highlighted that even in the absence of hydrogen sulfide, the characteristic aroma of scrambled chicken eggs can be best presented by only six odorants: (*E,Z*)-2,6-nonadienal, 2- and 3-methylbutanal (malty), 3-(methylsulfanyl)­propanal (cooked potato), 2,3-butanedione, and 1-octen-3-one. These findings provide a foundational framework for optimizing the aroma of plant-based egg alternatives.

## Graphical abstract

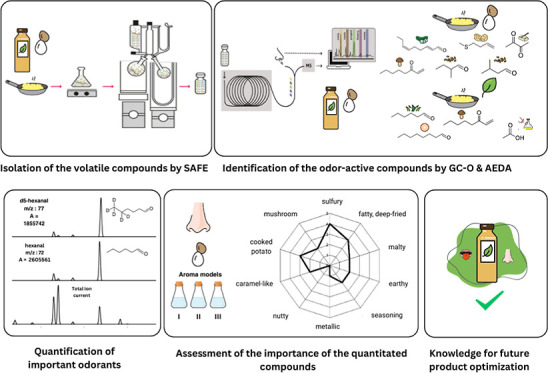

## Introduction

The plant-based food sector has been expanding rapidly over the past few years and is expected to grow further.^1^ The global retail sales of plant-based food in all categories have been reported to grow by 34% from 2019 to 2023.^2^ Among plant-based segments, egg analogs, with their 12% market share, are experiencing a 42% growth in sales, outpacing meat and dairy alternatives.^3^ As can also be seen with other alternative products, the shift to vegan eggs is driven by health-related concerns about the chicken eggs’ association with high cholesterol and allergenes.^4,5^ Ethical concerns about cage-based poultry farming and the environmental impact of egg production are also key motivations.^6,7^ Moreover, the ongoing shortage of chicken eggs in the US^8^ might accelerate the demand for plant-based alternatives. However, plant-based eggs as well as other vegan products still face resistance from consumers’ purchase decision because they are not yet closely mimicking flavors and appearance of conventional animal products.^9,10^ Therefore, closely replicating the organoleptic properties of cooked chicken eggs is essential for enhancing product appeal, subsequently being embraced and seamlessly integrated into consumers’ everyday diets. To achieve this, a comprehensive understanding of the aroma profiles of both cooked chicken eggs and egg analogs is crucial, allowing the identification of their flavor gap to better simulate the authentic egg experience. However, to date, no research has been conducted that directly addresses the flavor gap between animal- and plant-based eggs.

Raw eggs have a mild aroma in general, depending on the hen’s species and feeding routine, but different aroma levels were also reported during storage or cooking, due to chemical changes during storage.^11^ There were several attempts to analyze volatiles in cooked whole eggs, egg yolks, and egg whites separately,^12–18^ by using only gas chromatography (GC) and mass spectrum on combined gas chromatography–mass spectrometry (GC–MS). These studies revealed hundreds of compounds, in which aldehydes are the major volatiles, such as methylpropanal,^14,17^ 2-methylbutanal,^12,13,15,18^ and 3-methylbutanal.^15,17,18^ Still, the contribution of individual molecules to the overall egg aroma remains largely unexplored.

The Sensomics approach,^19^ which combines instrumental analysis with human odor perception, has been widely used to identify key flavor compounds in various foods.^20,21^ The process incorporates Gas Chromatography-Olfactometry (GC–O) combined with Aroma Extract Dilution Analysis (AEDA) to identify aroma-active compounds in food samples, followed by quantitation by Stable Isotope Dilution Assay (SIDA) to ensure precise measurement of odorants at trace levels.^19^ Odor activity values (OAVs)^22^ or Dose over Threshold factors (DoT factors)^23^ are then calculated to assess the significance of these compounds within the food matrix. The final step includes flavor reconstitution and omission experiments to identify the most critical compounds to the overall flavor perception.^19^ The Sensomics method has proven that only a small subset of volatile compounds significantly contributes to a food’s overall aroma profile.^19^

To date, the GC–O and AEDA approaches have been applied to study key odorants in the heated egg yolk,^24^ preserved egg yolk,^25^ and omelet,^26^ while the OAV/DoT concept has only been used in two studies. Moreover, only Tamura^26^ has used the SIDA and aroma reconstitution model to quantitate and confirm the contribution of 18 key compounds to the omelet aroma. The model comprised both odorants with OAVs > 1 (15 compounds) and OAVs ≤ 1 (3 compounds). Meanwhile, research on plant-based egg alternatives remains limited. Most studies have focused on chicken eggs only, leaving a substantial gap in understanding how well plant-based products replicate these aroma profiles. Furthermore, all previous research has involved the use of cooking oil in the preparation of scrambled egg and omelet samples,^15,16,26^ leaving the pure aroma profile of scrambled eggs without added oil unexplored. Analyzing samples without cooking oil is crucial, as the presence of oil can mask or alter key aroma compounds, thereby preventing an accurate assessment of the intrinsic odorant profile of the eggs.

To address these limitations, the present investigation aims to decode the odor gap between the scrambled chicken egg and commercial egg analogs by (i) identifying and screening the important odor-active compounds using GC–O and AEDA, (ii) quantitating selected odorants by GC–MS and GC–GC–MS (two-dimensional gas chromatography–mass spectrometry), using stable isotopically substituted odorants as internal standards, (iii) verifying the contribution of each odorant to the overall aroma profile by calculating DoT factors of the quantitated odorants for both animal-based and plant-based samples, and (iv) conducting aroma reconstitution for scrambled chicken eggs to recreate its aroma based on the identified key compounds, enabling a direct sensory evaluation and a better understanding of which compounds are most critical to the authentic scrambled egg aroma.

## Materials and Methods

### Chicken Egg and Vegan Egg Samples

Free-range chicken eggs were purchased from Migros (Wädenswil, Switzerland) and were 5 days old when being processed. The plant-based egg product was provided frozen by Lovely Day Foods GmbH (Berlin, Germany). The frozen sample was thawed at room temperature and shaken well prior to frying. The vegan product consists of water (74%), pea protein (12%), rapeseed oil (11.5%), and other ingredients (2.5%): gellan gum, table vinegar, salt, sodium citrate, carrot extract, paprika oil extract, and soy lecithin. The detailed nutrient compositions of both chicken egg and vegan egg products are provided in the supporting document (Table S4).

### Reference Odorants

The reference odorants acetic acid, 2,3-butanedione, 4-hydroxy-2,5-dimethyl-3­(2*H*)-furanone (furaneol), 4-methylphenol, (*E,Z*)-2,6-nonadienal, octanal, 2,3-diethyl-5-methylpyrazine, 2-methylbutanoic acid, 3-methylbutanoic acid, 2-(*E*)-nonenal, 1-octen-3-one, 2-ethyl-(3,5)-or-(3,6)-dimethylpyrazine, 3-hydroxy-4,5-dimethyl-2­(5*H*)-furanone (sotolon), hexanal, 3-(methylsulfanyl)­propanal (methional), 2-methylbutanal, 3-methylbutanal, 2′-aminoacetophenone, and 4-hydroxy-3-methoxybenzaldehyde were purchased from Sigma-Aldrich (Buchs, Switzerland); *trans*-4,5-epoxy-(*E*)-2-decenal and 2-acetyl-1-pyrroline (2-AP) were purchased from AromaLAB (Planegg, Germany). All reference odorants used were of analytical grade with a purity of at least 99%.

### Isotopically Substituted Standards

For quantitation, the following stable isotopically substituted standards were used: [^13^C_2_]-acetic acid (Sigma-Aldrich Chemie GmbH, Buchs, Switzerland), [^13^C_4_]-2,3-butanedione, [^2^H_5_]-(*E,Z*)-2,4-nonadienal, [^13^C_2_]-4-hydroxy-2,5-dimethyl-3­(2*H*)-furanone, [^2^H_4_]-octanal, [^2^H_2_]-*trans*-4,5-epoxy-(*E*)-2-decenal, [^2^H_7_]-2,3-diethyl-5-methylpyrazine, [^2^H_9_]-3-methylbutanoic acid, [^2^H_2_]-(*E*)-2-nonenal, [^13^C_5_]-2-AP, [^2^H_3_]-1-octen-3-one, [^2^H_5_]-2-ethyl-3,6-dimethylpyrazine, [^2^H_2_]-4-methoxyphenol, [^13^C_2_]-3-hydroxy-4,5-dimethyl-2­(5*H*)-furanone, [^2^H_5_]-hexanal, [^2^H_3_]-3-(methylsulfanyl)­propanal, [^2^H_3_]-2-methylbutanal, and [^2^H_2_]-3-methylbutanal (AromaLAB GmbH, Martinsried, Germany).

### Other Chemicals and Materials

Diethyl ether (Merck KGaA) was freshly distilled before use. Anhydrous sodium sulfate was purchased from Carl Roth (Roth AG, Arlesheim, Switzerland). Propionic acid was purchased from Sigma-Aldrich (Buchs, Switzerland). Neutral Miglyol 812 was purchased from Hänseler AG (Herisau, Switzerland). Kala Namak (Vitam, Germany) salt and pure gelatin powder (Dr. Oetker, Germany) were purchased from Migros (Wädenswil, Switzerland). The pea protein powder was kindly supplied by Lovely Day Foods GmbH (Berlin, Germany).

Certified methanethiol (0.2% in toluene) and hydrogen sulfide (H_2_S) (0.2% in toluene) standards were purchased from SwissLabs (Swiss Laboratories Chromatography Services SAS, Mulhouse, France) for quantitation. Due to the unavailability of high purity methanethiol and H_2_S in Switzerland, these compounds could not be used for the preparation of the aroma reconstitution models.

### Preparation of Scrambled Chicken Eggs and Scrambled Vegan Eggs

A Teflon-coated pan was preheated to 120 **°**C prior to every cooking. The temperature was checked by a digital thermometer (Amarell, Germany). For chicken eggs, two eggs (105–108 g) were cracked and hand-whipped until homogenized. The mixture was then poured in the preheated pan and cooked for 2 min with continuous agitation. The plant-based egg product was shaken well before sampling, and then 100 g of vegan egg liquid was weighed and cooked for 3–4 min with continuous agitation until a similar texture to the chicken scrambled egg was created. The chosen cooking process for chicken eggs was based on previous studies^16,26^ describing laboratory experiments in a comparable context. The preparation of the plant-based egg followed the recommended method on the product’s label to achieve a texture comparable with chicken-scrambled eggs. Samples were frozen with liquid nitrogen instantly after cooking to avoid losses of volatiles and subsequently ground finely using a laboratory miller (Carl Roth, GmbH + Co. KG, Karlsruhe), thus obtaining powdered samples for further analysis.

### Gas Chromatography-Olfactometry and Aroma Extract Dilution Analysis

For the screening of important odor-active compounds, each 50 g of the grounded scrambled chicken egg and grounded scrambled vegan egg were weighed in 250 mL Erlenmyer flasks. The samples were extracted with 500 mL of diethyl ether by vigorous stirring with a magnetic stirrer (IKA-Werke GmbH & Co. KG, Staufen) at room temperature for at least 12 h to enable the efficient extraction of volatile compounds. During extraction time, the flasks were sealed with stoppers and covered with aluminum foil. After extraction, the diethyl ether phase was filtered through filter paper (185 mm, Whatman, Germany), then directly subjected to solvent-assisted flavor evaporation (SAFE) distillation with instrumental settings as previously described.^27^ The thawed distillates were dehydrated using anhydrous sodium sulfate and then concentrated on a Vigreux column to 5 mL. The distillates were then transferred to graduated concentrator tubes (provider) and further reduced to a final volume of 300 μL, under a gentle stream of nitrogen.

Performance of GC–O analysis on samples at a flavor dilution factor (FD factor) 1 was carried out by three experienced panelists. GC–O in combination with AEDA analysis was performed by one experienced panelist on both animal and plant-based samples, in the same manner, using the same parameters as described in a previous study.^27^

### Quantitation of Odorants by Stable Isotope Dilution Assay (SIDA)

To account for the anticipated presence of trace volatiles, multiple sample sizes were extracted to ensure that sufficient quantities of the targeted odorants were available. Specifically, 5, 20, 50, and 100 g of cooked samples were added with 50, 200, and 500 mL (for both 50 and 100 g samples) of distilled diethyl ether in Erlenmeyer flasks, respectively. Stable isotopically substituted odorant standards were incorporated in the sample in quantities consistent with the anticipated levels for the target compounds (0.04–3313 μg). The sample was stirred for at least 12 h with a magnetic stirrer, followed by the separation of the diethyl ether phase by means of a filtration funnel, and then subjected to SAFE analysis followed by concentration as previously described to obtain the final 300 μL of extracts. The aroma of each 300 μL extract was then quickly assessed using sniffing paper to ensure that it represented the aroma of the sample before extraction.

Depending on the target compound, the quantitation was done with either a GC–MS or GC–GC–MS system in the electron ionization or positive chemical ionization (EI or PCI) mode. With GC–MS and GC–GC–MS systems in the EI mode, the instrumental settings were both described in a previous study.^27^ For those compounds quantitated with GC–GC–MS in the PCI mode, the same system and temperature program as the EI mode were used with a few modifications. The ion source temperature was set at 180 °C instead, and methane was used as the reactant gas. The MS was also operated in the selected ion monitoring (SIM) mode with individual quantifier ions for the analyte and standard of each target compound. Details of targeted odorants and their corresponding quantitation methods are provided in the Supporting Information (Table S1).

### Quantitation of Acetic Acid in Plant-Based Samples by GC–FID

Given the expected abundance of acetic acid in the vegan egg product based on AEDA results (Table 1) and its presence in the product’s original recipe, the quantity of this compound was analyzed by a Gas Chromatography (GC) system (Thermo Trace GC Ultra) with Flame Ionization Detection (FID). The scrambled vegan egg powder (1 g) was weighed into a plastic centrifuge tube (15 mL), followed by an addition of 5 mL of diethyl ether and 1 mL ultra-pure water. Propionic acid (1030 μg) was incorporated as an internal standard corresponding to the expected acetic acid content. Extraction was performed for 30 min using an overhead shaker, followed by centrifugation at 4000 rpm (3220 g) for 15 min (Eppendorf AG, Hamburg, Germany). The resulting supernatant was utilized for subsequent quantitation with the GC–FID system, which has been described before.^27^ For calibration, four different concentrations of acetic acid were each prepared in a mixture composed of 5 mL of diethyl ether, 1 mL of distilled water, and 1 g of simulation solution (12% pea protein, 88% ultra-pure water) of the original plant-based egg product, and the sample amount of propionic acid (1030 μg), as in the samples. All calibration solutions were then extracted by the same procedure as described above and subjected to GC–FID analysis. The Supporting Information provides details on the linear regressions used for quantitation (Table S2).

**Table 1:** Odor-Active Compounds Perceived during AEDA

			retention index on		FD factor^d^	
no^a^	odorants^b^	odor quality^c^	DB-FFAP	DB-5	RE	VE
1	2-methylbutanal	malty	915	712	64	64
2	3-methylbutanal	malty	917	710	64	64
3	2,3-butanedione	butter-like	944	612	16	
4	hexanal	green, grassy	1072	792	64	64
5	octanal	green	1278	1002	32	64
6	1-octen-3-one	mushroom-like	1295	977	128	128
7	2-acetyl-1-pyrroline (2-AP)^e^	roasty, popcorn-like	1324	917	32	
8	acetic acid	vinegar-like	1436	618		1024
9	2-ethyl-3,6-dimethylpyrazine	earthy	1436	1060	8	
10	2-ethyl-3,5-dimethylpyrazine	earthy	1440	1073	8	
11	3-(methylsulfanyl)propanal	cooked potato	1450	899	1024	64
12	2,3-diethyl-5-methylpyrazine	earthy, roasty	1481	1155	8	
13	(*E*)-2-nonenal	cardboard-like, fatty	1529	1168	16	
14	(*E,Z*)-2,6-nonadienal	cucumber-like	1579	1159	128	
15	2-methylbutanoic acid^f^	sweaty, cheesy	1659	867	16	1024
16	3-methylbutanoic acid^f^	sweaty, cheesy	1659	861	16	1024
17	*trans*-4,5-epoxy-(*E*)-2-decenal	metallic-like	2020	1377		128
18	4-hydroxy-2,5-dimethyl-3(2*H*)-furanone	caramel-like	2025	1070	32	
19	4-methylphenol (*p*-cresol)	smoky, horse stable-like	2080	1078	64	
20	3-hydroxy-4,5-dimethyl-2(5*H*)-furanone (sotolon)	seasoning	2210	1105		64

a Odorants were numbered according to their retention indices on capillary column FFAP.

b Odorant identified by comparison of its odor quality and intensity at the sniffing port and retention indices on capillaries DB-FFAP, DB-5, as well as mass spectra (EI) with data of reference compounds.

c Odor quality perceived at the sniffing-port.

d Flavor dilution factor determined by AEDA on capillary FFAP.

e No unequivocal mass spectrum was obtained, the identification is based on the remaining criteria in footnote b.

f Compound has not been reported in cooked chicken eggs yet.

### Semi-Quantitation of H_2_S and Methanethiol

H_2_S and methanthiol were semiquantitated with head space-gas chromatogram-orbitrap-mass spectrometry. Amounts of 2 g of the scrambled chicken egg and cooked vegan egg (cooking as previously described) were placed directly after cooking into a 20 mL SPME vial. Measurements were started immediately after the sample was weighed into the SPME vial. The system used was a Trace 1310 gas chromatograph (with SSL injector with split liner without glass wool) coupled with an Orbitrap Exploris 240 MS with 60 K resolution (both from Thermo Fisher Scientific, Brechbühler, Schlieren, Switzerland). The injections were realized with a Triplus RSH Autosampler in a headspace configuration, whereby the syringe was preheated at 80 °C and the injected volume was 1 mL. Samples were incubated at 60 °C for 10 min under constant agitation (750 rpm). The inlet temperature was set at 150 °C and the split flow at 20 mL/min. The separation of the volatiles was performed on the capillary column Thermo VVOC B 60 m × 0.32 mm 5 μm using helium as a carrier gas at a constant flow of 1.2 mL/min by the application of the following temperature program: 40 °C for 5 min, then raised by 5 °C/min to 100 °C, then raised again by 15 °C/min to 180 °C, and held for 1 min. The transfer line between the GC oven and Orbitrap was set at 220 °C. The measurements were done in the electron ionization (EI) mode (70 eV) using the full scan mode, the temperature of the source was set at 240 °C. Quantitation was done by extracting ions determined during pre-experiments (*m*/*z* 33.98710, *m*/*z* 31.97150, *m*/*z* 32.97930, and *m*/*z* 35.98295 for H_2_S and *m*/*z* 46.99500, *m*/*z* 48.00280, and *m*/*z* 44.97930 for methanethiol). The Chromeleon 7.3.2 chromatography data system was used to control the system and treat the obtained data.

External 3-point calibrations were used to calculate the concentrations of both analytes in the samples (Supporting Information, Table S2). Both certified standards were diluted in 2 mL toluene and measured in the same way as the samples. Peak areas were recorded.

### Dose over Threshold Factors

The quantitated concentrations of target odorants in the scrambled chicken egg and scrambled vegan egg were divided by the odor threshold (OT) values of each compound in water to derive dose over threshold factors (DoT factors). This concept has been previously applied for cocoa and vanilla in other studies.^23,27^ The data was presented in Table 3 and visualized in Figure 1.

**Figure 1:**
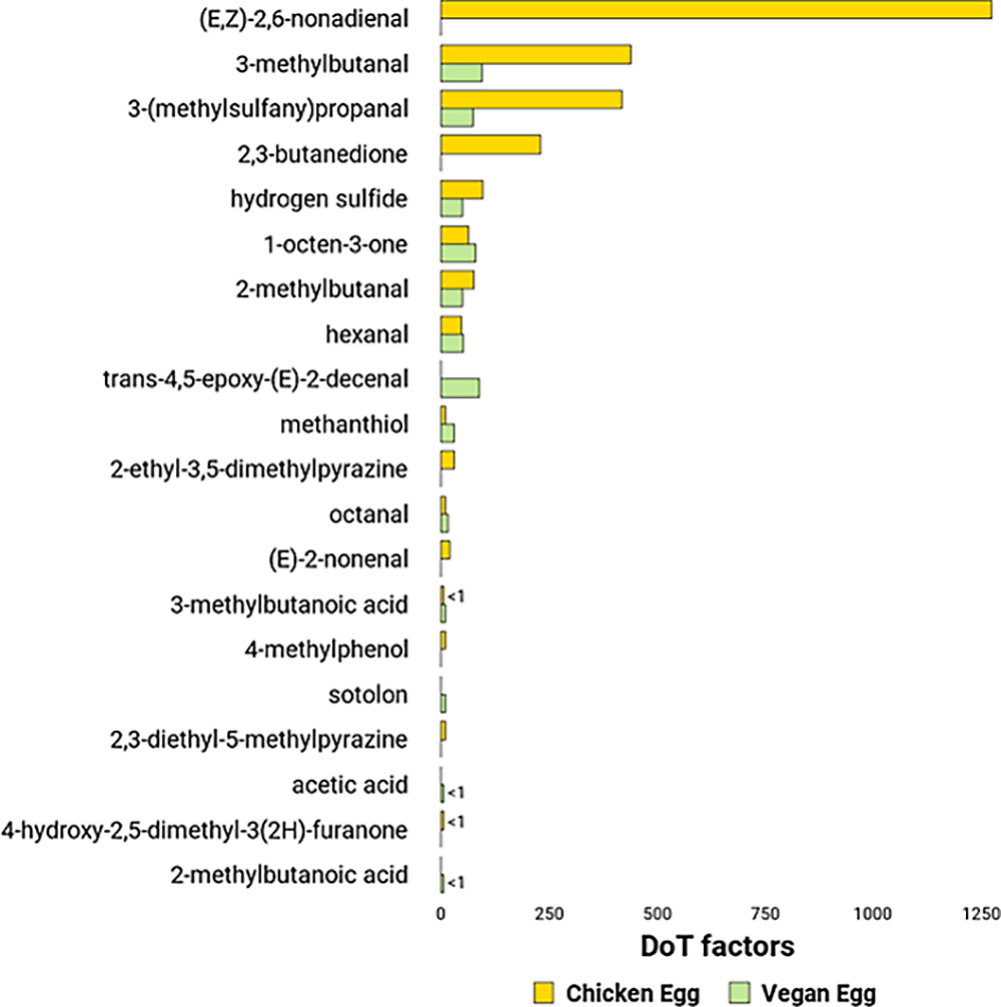
Comparison of the Dose over Threshold factors (DoT factors) of selected odorants in the scrambled chicken egg with the scrambled vegan egg.

### Aroma Reconstitution Experiment

For aroma reconstitution, the model system consisted of a protein-oil-water matrix and 11 aroma-active compounds of the scrambled chicken egg with DoT factor >1 at the quantitated concentrations. To imitate the matrix of the scrambled chicken egg, gelatin, Miglyol, and deionized water were combined at a weight ratio of 12:12:76, based on the previously published contents of main constituents (protein–oil–water ratio) analyzed in the cooked chicken egg.^28,29^ The stock solution of each odorant was prepared by dissolving the known amount of pure substances in 10 mL Miglyol except for H_2_S, which was substituted with Kala Namak salt due to the high volatility of H_2_S and the associated risk of significant losses during handling.^30^ The concentration of H_2_S in the salt (28,351 μg/kg) was determined by preparing a 12% salt solution in deionized water and measured in the same manner and instrumental setting as described earlier for the chicken egg and vegan egg samples.

To prepare the reconstitution model, the matrix mixture was made in advance, stirring vigorously with moderate heating at 30 °C until the gelatin was well dissolved. While still in liquid form, 5 g of the matrix mixture was weighed in a 100 mL Erlenmeyer flask. Eleven compounds were pipetted directly from their stock solutions into the matrix in quantities determined from quantitative results, adjusted for the 5 g matrix: (*E,Z*)-2,6-nonadienal (0.03 μg), 3-methylbutanal (1.1 μg), methional (0.9 μg), 2,3-butanedione (1.15 μg), 2-methylbutanal (0.57 μg), 1-octen-3-one (0.01 μg), hexanal (0.56 μg), 2-ethyl-3,5-dimethylpyrazine (0.04 μg), (*E*)-2-nonenal (0.02 μg), octanal (0.06 μg), and 4-methylphenol (0.07 μg). As for H_2_S, 170 mg of Kala Namak was used to achieve the corresponding required amount of H_2_S (4.84 μg) for 5 g of matrix. The aroma model mixture was then sealed and left for 1 h to stabilize before proceeding with the evaluation as mentioned in the section on sensory evaluation.

### Omission Experiment

To evaluate the contribution of certain aroma compounds to the overall flavor profile of the scrambled chicken egg, two more aroma models RM1 and RM2 were prepared in the same manner as described earlier. Details of the odorant composition of each model were described in Table 4. The experimental design aligns with established approaches used in studies on various food products.^21,31,32^

By systematically excluding specific compounds, the study aimed to differentiate between compounds that were essential to the aroma and those that played a secondary role.^31^

### Sensory Evaluation

The sensory evaluation of the aroma reconstitution models was conducted in the sensory laboratory at the Zurich University of Applied Sciences (ZHAW) under controlled conditions, following a consensus profile according to DIN EN ISO 13299.^33^ Six assessors (all female, between 30 and 45 years old) evaluated the aroma reconstitution models. The assessors were informed about the research project’s objectives prior to the study and recruited from ZHAW on a voluntary basis. All assessors were experienced with sensory evaluations and familiar with the testing method, as well as with the product category. Prior to evaluation, assessors participated in a training session where they were familiarized with ten selected aroma descriptors, which were demonstrated by reference compounds prepared in Miglyol at a concentration 200-fold above their odor threshold in oil (Supporting Information, Table S3), ensuring an intense perception of the selected references with their specific attributes. Those ten odor attributes were represented by given compounds in parentheses, except for the last descriptor: mushroom-like (1-octen-3-one), cooked potato-like (methional), fatty ((*E*)-2-nonenal), malty (3-methylbutanal), earthy (2-ethyl-3,5-dimethylpyrazine), caramel-like (4-hydroxy-2,5-dimethyl-3­(2*H*)-furanone), seasoning-like (sotolon), metallic (*trans*-4,5-epoxy-(*E*)-2-decenal), and nutty (2-acetyl-2-thiazoline). For the descriptor “sulfury” 10% w/w Kala Namak salt in distilled water was prepared for demonstration due to the unavailability of H_2_S. After training, the assessors evaluated the orthonasal odors of the aroma reconstitution models by rating the intensity of these attributes on a six-point categorical scale, ranging from 0 (“not perceivable”) to 5 (“very intense”). After individual ratings, the final intensity values for the different attributes were determined by consensus through discussion. The results were presented in spider plots (Figure 2).

**Figure 2:**
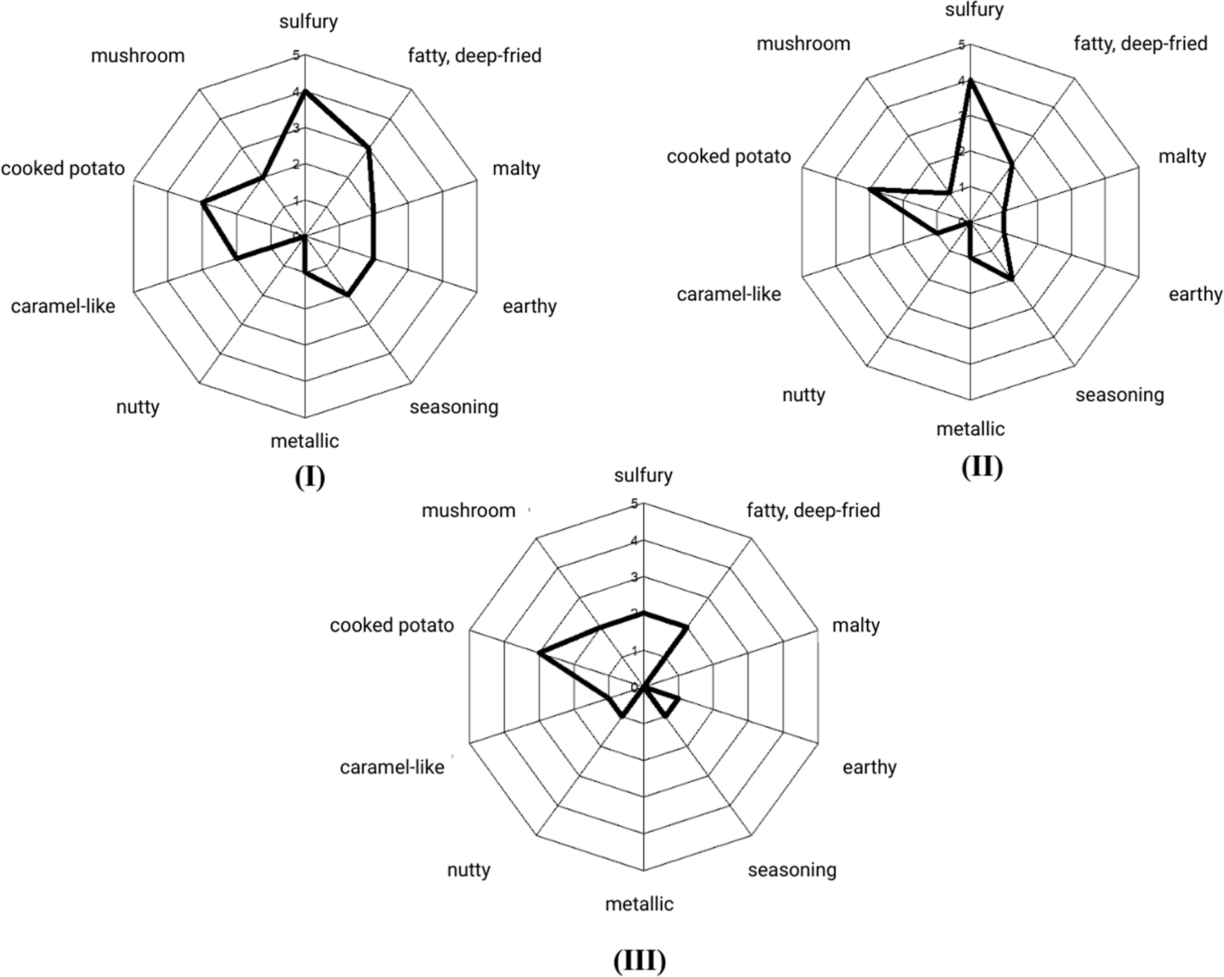
Aroma profile analysis of reconstitution model RM 1 (**I**) as the complete model, and the simplified models RM 2 (**II**) and RM 3 (**III**) using a trained panel. The molecular compositions are listed in Table 4.

In further evaluations, conducted in the style of a consumer-study setting, the association and resemblance of the aroma reconstitution models to scrambled eggs were evaluated. The study was carried out in the sensory laboratory at the ZHAW under controlled conditions. A total of 24 participants took part in this test. The participants were informed about the research project’s objectives prior to the study and recruited on a voluntary basis from staff members and students of ZHAW. None of the 24 participants were involved in the project or had prior training in recognizing specific egg dish odor impressions. The three aroma reconstitution models (10 g) were presented to the participants in 100 mL Erlenmeyer flasks with glass stoppers, blinded by labeling with random three-digit codes. The aroma reconstitution models were served sequential monadic, following a randomized presentation design (Williams Latin Square). Tablets were provided on-site, and data were collected using Fizz Collect software (SARL Biosystèmes, Version 3.9.0, Couternon, France). The data acquisition process was completely anonymous, and sociodemographic data (i.e., age group, gender, type of diet, and frequency of consumption of hen’s egg) were collected from all participants at the end of the test. For each aroma reconstitution model, participants were asked to (1) answer the question “What food does the sample remind you of?” by associating the aroma with predefined categories: scrambled egg, egg yolk, omelet, boiled egg, fried egg, and others, (2) rate how closely each aroma reconstitution model resembled scrambled chicken egg aroma using a five-point scale ranging from “very well-perceivable” to “unable to assess”. The testing procedure lasted approximately 10 min and included an additional question in which the participants were asked to describe the odor with their own words (results are not reported in this paper). The collected data were analyzed quantitatively (Figure 3).

**Figure 3:**
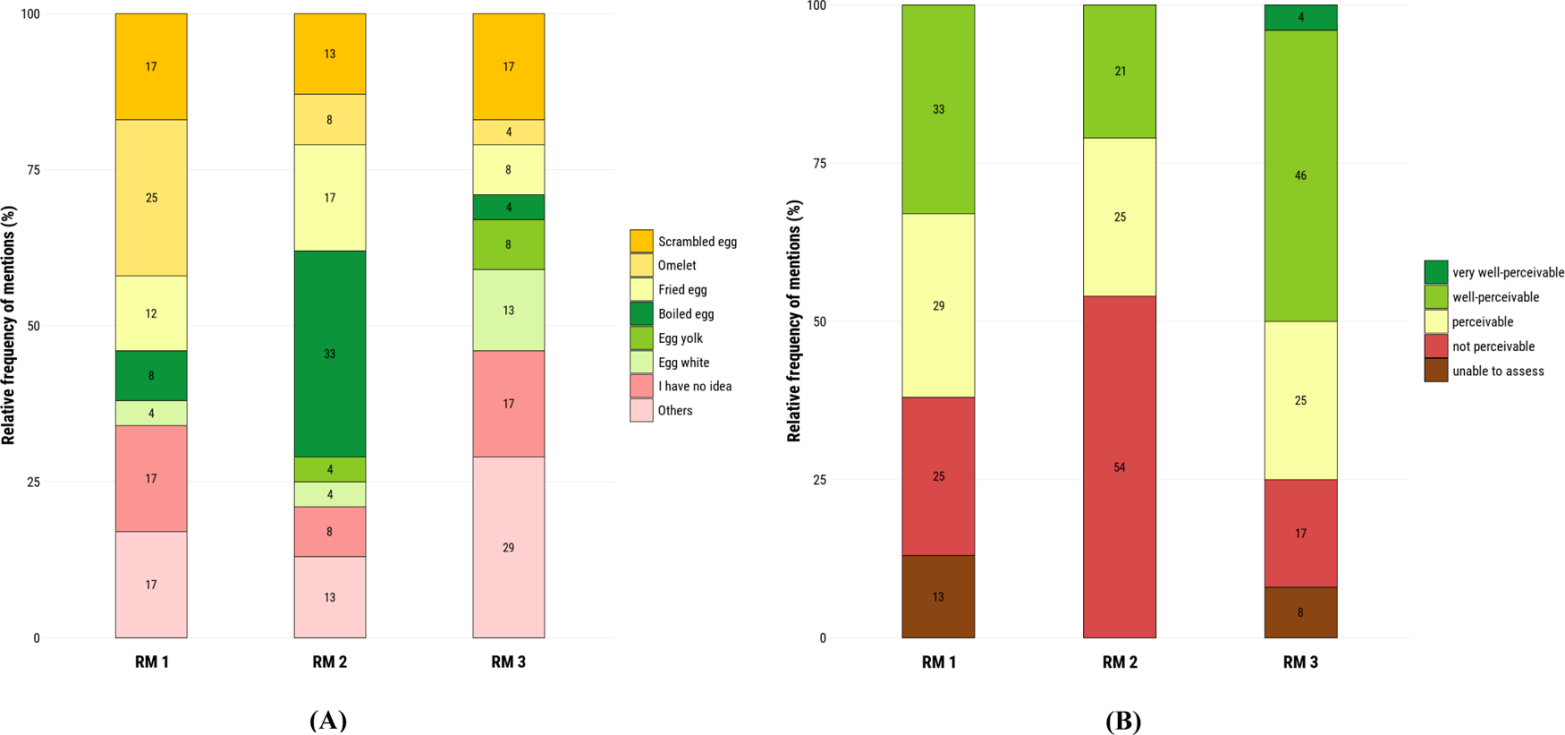
Sensory evaluation of aroma reconstitution models (*n* = 24) (A) degree of associations between aroma reconstitution models and egg-related dishes. (B) Degree of resemblance of the aroma reconstitution models to the scrambled chicken egg. The molecular compositions of aroma models are listed in Table 4.

### Statistical Analysis

Statistical analysis and data visualization were performed with RStudio (version 4.3.3, Posit PBC).

## Results and Discussion

### Identification of Odor-Active Compounds in Scrambled Chicken Eggs

In general, the AEDA revealed a total of 20 odor-active compounds in both animal- and plant-based samples, with FD factors ranging from 8 to 1024, as presented in Table 1.

Regarding the aroma profile of scrambled chicken eggs, there were 17 important odor-active compounds identified with high flavor dilution (FD) factors. The compound 3-(methylsulfanyl)-propanal (methional), characterized by a cooked potato-like odor, exhibited the highest FD factor (FD 1024). This was followed by the cucumber-like odorant (*E,Z*)-2,6-nonadienal (FD 128) and mushroom-like 1-octen-3-one (FD 128). These compounds demonstrated high FD factors comparable to the findings from studies on omelets^26^ and cooked egg yolk.^24^ Additionally, other compounds previously identified in omelets were also detected in scrambled chicken eggs, albeit with lower FD factors: octanal (citrus-like, FD 32), 2,3-butanedione (buttery, FD 64), *trans* **-**4,5-epoxy-(*E*)-2-decenal (metallic-like, FD 64), 2-AP (popcorn-like, FD 16), (*E*)-2-nonenal (fatty, FD 32), and 4-hydroxy-2,5-dimethyl-3­(2*H*)-furanone (caramel-like, FD 32). Despite their relatively lower FD factors in this study, these compounds were confirmed as contributors to the scrambled egg aroma.

At lower FD factors (FD 16), there were three earthy smelling compounds, including 2-ethyl-3,6-dimethylpyrazine, 2-ethyl-3,5-dimethylpyrazine, and 2,3-diethyl-5-methylpyrazine. These alkylpyrazines were all previously found as volatiles in cooked whole chicken eggs.^14^ They are formed via Maillard-type reactions in the presence of specific amino acids.^34^ Interestingly, 2-methylbutanoic acid (FD 16) and 3-methylbutanoic acid (FD 16) were characterized for the first time as volatile constituents of cooked chicken eggs.

To ensure that no significant odor-active compounds were overlooked, additional odorants previously identified in omelet studies^26^ with high FD factors, namely, 2′-aminoacetophenone (foxy, FD 512), 4-hydroxy-3-methoxybenzaldehyde (vanilla-like, FD 256), and γ-(*Z*)-6-dodecenolactone (peach-like, FD 256), were also examined. Verification for the first two compounds was conducted by comparing their odor qualities at the sniffing port, retention indices on DB-FFAP columns, and mass spectra against reference compounds. For γ-(*Z*)-6-dodecenolactone, due to the unavailability of a reference compound, only its mass fragments were analyzed by GC–MS. However, none of these odorants could be confirmed in scrambled chicken egg samples.

### Identification of Odor-Active Compounds in Scrambled Vegan Eggs and Comparison with Scrambled Chicken Eggs

The solvent extract of the plant-based egg exhibited a strong acidic and malty aroma when sniffed on a strip of filter paper, in comparison with the extract of the scrambled chicken egg, which elicited an intense egg-like odor. The structural identification of odor-active compounds was performed using the same methodology as that for scrambled chicken eggs. A total of 11 odor-active compounds were identified in the vegan egg sample, with FD factors ranging from 8 to 1024 (Table 1). The most intense contributors to the aroma were acetic acid (vinegar-like, FD 1024) and the short-chain-branched acids, 2-methylbutanoic acid and 3-methylbutanoic acid (sweaty, FD 1024). Their high intensity aligns with the use of plant-based ingredients in the vegan product, including pea protein and soybean lecithin, which are rich in amino acid precursors.^35,36^

Other important contributors to the vegan egg aroma included 1-octen-3-one (mushroom-like, FD 128), *trans*-4,5-epoxy-(*E*)-2-decenal (metallic-like, FD 128), octanal (green, FD 64), and 4-hydroxy-2,5-dimethyl-3­(2*H*)-furanone (caramel-like, FD 32). While *trans*-4,5-epoxy-(*E*)-2-decenal was previously reported as a key odorant derived from soybean lecithin,^35^ octanal, and 1-octen-3-one are likely formed by the oxidation of unsaturated fatty acids such as linoleic acid and oleic acid, commonly found in plant-based oils.^37,38^ A caramel-like compound, 4-hydroxy-2,5-dimethyl-3­(2*H*)-furanone, is generated via Maillard-type reactions of plant-based proteins and reducing sugars during heating. In addition, the presence of 3-hydroxy-4,5-dimethyl-2­(5*H*)-furanone (sotolon) (seasoning, FD 64) was reported earlier in the plant-based hydrolyzed protein^37^ and rapeseed oil,^39^ which are ingredients in the vegan egg product.

When compared with scrambled chicken eggs (RE column), eight shared odor-active compounds were identified, with differences in FD factors reflecting their distinct aroma profiles. Scrambled chicken eggs exhibited a higher FD factor for cooked potato-like compound methional (FD 1024 vs. 64 in the vegan egg), and the following odorants were exclusively found in the chicken egg sample, i.e., (*E,Z*)-2,6-nonadienal (cucumber-like), 2,3-butanedione (butter-like), 2-AP (roasty, popcorn-like), three alkylpyrazines (earthy), (*E*)-2-nonenal (fatty), furaneol (caramel-like), and *p*-cresol (smoky, paint-like). In contrast, the vegan egg product was enriched in acetic acid (FD 1024), which was used as a pH regulator, and in the seasoning-like compound sotolon (FD 64). Moreover, the plant-based sample also exhibited higher FD factors of 2- and 3-methylbutanoic acids (both FD 1024 vs 16 in scrambled chicken eggs), contributing to its dominant acidic and sweaty aroma.

### Quantitation of Selected Important Odor-Active Compounds in Scrambled Chicken Eggs and Scrambled Vegan Eggs

Among 20 odorants characterized by AEDA, 15 odorants of the scrambled chicken egg and 11 odorants of the scrambled vegan egg were quantitated by means of SIDA (Table 2). Although H_2_S and methanethiol were not identified during the GC–O and AEDA procedures due to their high volatility, their presence have been reported as important contributors to cooked chicken egg aroma in previous studies.^24,26^ Therefore, their concentrations were also determined in the current study, as shown in Table 2.

**Table 2:** Concentrations of Important Odor-Active Compounds in the Scrambled Chicken Egg (RE) and Scrambled Vegan Egg (VE)

		concentration^a^ (μg/kg)	
odorants	odor quality	RE	VE
hydrogen sulfide	boiled-egg like	967	498
2,3-butanedione	butter-like	230	nd
3-methylbutanal	malty	220	47
3-(methylsulfanyl)propanal (methional)	cooked potato	180	32
2-methylbutanal	malty	113	75
hexanal	green, grassy	112	122
3-methylbutanoic acid	sweaty, cheesy	66	1761
4-hydroxy-2,5-dimethyl-3(2*H*)-furanone	caramel-like	34	nd
2-methylbutanoic acid	sweaty, cheesy	24	850
4-methylphenol (*p*-cresol)	smoky, horse stable-like	14	nd
octanal	green	12	58
2-ethyl-3,5-dimethylpyrazine	earthy	8.3	nd
(*E*,*Z*)-2,6-nonadienal	cucumber-like	5.7	nd
methanethiol	sulfury	5.6	18
(*E*)-2-nonenal	cardboard-like, fatty	3.8	nd
1-octen-3-one	mushroom-like	1.0	1.3
2,3-diethyl-5-methylpyrazine	earthy	0.04	nd
2-ethyl-3,6-dimethylpyrazine	earthy	nd	nd
*trans*-4,5-epoxy-(*E*)-2-decenal	metallic-like	nd	19
3-hydroxy-4,5-dimethyl-2(5*H*)-furanone (sotolon)	seasoning	nd	2.8
acetic acid	vinegar-like	nd	3393
2-acetyl-1-pyrroline	roasty, popcorn-like	nd	nd

a Mean values of triplicates with a relative standard deviation < 15%. nd: not detected.

The results obtained for animal-based samples shows that H_2_S exhibited the highest concentration at 967 μg/kg, followed by 2,3-butanedione (230 μg/kg), 3-methylbutanal (220 μg/kg), methional (180 μg/kg), 2-methylbutanal (113 μg/kg), and hexanal (112 μg/kg). These compounds, which were previously reported in the omelet,^26^ highlight a consistent trend in the generation of important odor-active compounds across different cooked egg products. Meanwhile, 2,3-butanedione was quantitated in the scrambled chicken egg at higher concentrations compared to the omelet^26^ (230 μg/kg vs 33 μg/kg). A previous study has shown that 2,3-butanedione is indirectly formed as a result of valine and isoleucine anabolism,^40^ both of which are present in egg proteins. This study also mentioned that environmental factors, such as the temperature, pH value, and substrate availability play critical roles in diacetyl formation. Hence, the thermal conditions during cooking, such as high temperatures and exposure to air during the preparation of scrambled chicken eggs, can promote nonenzymatic reactions, including the decarboxylation of these amino acids. Alternatively, Maillard-initiated sugar degradation at elevated temperatures also results in diacetyl formation.^41^ The cooking method for scrambled eggs, which involves continuous agitation and uniform heat exposure, may, therefore, promote greater diacetyl formation compared to that of omelets. Other compounds, on the contrary, were only present in trace amounts, such as 4-hydroxy-2,5-dimethyl-3­(2*H*)-furanone (34 μg/kg), (*E,Z*)-2,6-nonadienal (5.7 μg/kg), 1-octen-3-one (1.0 μg/kg), 4-methylphenol (14 μg/kg), 2-ethyl-3,5-dimethylpyrazine (8.3 μg/kg), and 2,3-diethyl-5-methylpyrazine (0.04 μg/kg). The first three odorants were found in omelets in comparable quantities, confirming their contribution to the scrambled chicken egg aroma. Meanwhile, the three latter odorants were only qualitatively identified in whole chicken eggs by GC–MS.^14^ The generation of 4-methylphenol (*p*-cresol) might originate from the thermal degradation of tyrosine in whole chicken eggs.^42^

Notably, two short-chain branched acids were detected in scrambled chicken eggs for the first time, i.e., 2-methylbutanoic acid (24 μg/kg) and 3-methylbutanoic acid (66 μg/kg). Their presence suggests that the degradation of free amino acids, particularly leucine, plays a role in scrambled egg aroma generation.^43,44^ As an example, leucine may react to 3-methylbutanal through the Strecker reaction or directly to the acid under oxidative conditions in the presence of oxygen and transition metals.^45^ Their absence in omelets could be due to differences in precursor availability or thermal conditions, as these acids are often formed at moderate cooking temperatures and may degrade under prolonged heating.^46^

The quantitation of 2-AP in scrambled chicken eggs presented significant challenges due to its inherently low concentration in the matrix, despite the fact that it was detected during AEDA with a dilution factor of 32. Analyses using GC–GC–MS in both electron ionization (EI) and CI modes were conducted to enhance sensitivity and specificity. A total of 50 g of the sample matrix was spiked with 0.04 μg of a corresponding labeled standard (0.8 μg/kg). This standard was reliably detected, suggesting that the limit of quantitation for 2-AP lies at or above this level. This difficulty can be attributed to the well-documented chemical instability of 2-AP, as it is prone to degradation during storage and sample preparation.^47–49^ By comparison, a previous study on omelets successfully quantitated 2-AP at 0.31 μg/kg, thus suggesting its lower concentration in scrambled chicken eggs. Additionally, the higher FD observed for 2-AP in omelets (FD 256) compared to scrambled chicken eggs (FD 32) further supports its reduced concentration as well as contribution to the scrambled chicken egg aroma. This disparity likely arises from differences in thermal generation and matrix retention, with the cooking temperature being lower and the moisture content being higher for scrambled chicken eggs, thus limiting 2-AP formation and stability.

Regarding scrambled vegan eggs, 13 important odor-active compounds were quantitated by SIDA, with their concentrations shown in Table 2. The results were well in alignment with the AEDA findings, in which acetic acid exhibited the highest concentration at 3393 μg/kg, followed by 3-methylbutanoic acid (1761 μg/kg) and 2-methylbutanoic acid (850 μg/kg). This difference suggests a higher abundance of leucine and isoleucine precursors in the vegan formulation, potentially due to the composition of the pea protein isolates. H_2_S and methanethiol, two compounds with sulfury notes, were detected at 498 μg/kg (vs 967 μg/kg in chicken eggs) and 18 μg/kg (vs 5.6 μg/kg in chicken eggs) in the vegan egg, respectively. Lipid oxidation products, including hexanal and octanal, were present at comparable levels in both matrices.

Interestingly, the vegan egg exhibited lower levels of 2-methylbutanal (75 μg/kg) and 3-methylbutanal (47 μg/kg) compared to the chicken egg (113 and 220 μg/kg, respectively). These Strecker aldehydes are formed through the thermal degradation of amino acids, and their reduced concentration in vegan eggs suggests differences in precursor availability or reactivity. Additionally, 4-hydroxy-2,5-dimethyl-3­(2*H*)-furanone, a compound linked to sugar degradation via Maillard-type reactions, was quantitated at only 2 μg/kg in the vegan egg compared to 34 μg/kg in the chicken egg. This indicates a lower extent of Maillard reaction activity, likely due to reduced sugar and amino acid content in the plant-based matrix, which limits the formation of this compound during cooking.

### Dose over Threshold Factors (DoT Factors)

To gain a deeper understanding of the aroma profiles of the scrambled chicken egg and scrambled vegan egg, dose over threshold factors (DoT factors), calculated as the ratio of concentration to odor threshold in water, were determined for each quantitated compound. The DoT factors provide insights into the contribution of individual odorants to the overall aroma (Table 3 and Figure 1).

**Table 3:** Orthonasal Odor Thresholds (OTs) and Dose over Threshold Factors (DoT factors) of Important Odor-Active Compounds in the Scrambled Chicken Egg (RE) and Scrambled Vegan Egg (VE)

		DoT factor^b^	
odorants	OT^a^ (μg/kg)	RE	VE
(*E*,*Z*)-2,6-nonadienal	0.0045	1274	nd
3-methylbutanal	0.5	439	94
3-(methylsulfanyl)propanal (methional)	0.43	418	74
2,3-butanedione	1	230	n.a
hydrogen sulfide	10	97	50
2-methylbutanal	1.5	75	50
1-octen-3-one	0.016	63	79
hexanal	2.4	47	51
2-ethyl-3,5-dimethylpyrazine	0.28	30	3.8
(*E*)-2-nonenal	0.19	20	n.a
methanthiol	0.59	10	30
4-methylphenol (*p*-cresol)	3.9	3.7	n.a
octanal	3.4	3.5	17
2,3-diethyl-5-methylpyrazine	0.031	1.2	n.a
4-hydroxy-2,5-dimethyl-3(2*H*)-furanone	53	<1	n.a
3-methylbutanoic acid	490	<1	3.6
2-methylbutanoic acid	3100	<1	<1
2-ethyl-3,6-dimethylpyrazine	25	n.a	n.a
*trans*-4,5-epoxy-(*E*)-2-decenal	0.22	n.a	88
3-hydroxy-4,5-dimethyl-2(5*H*)-furanone (sotolon)	1.7	n.a	1.7
acetic acid	5600	n.a	<1
2-acetyl-1-pyrroline	0.053	n.a	n.a

a Odor threshold value in water according to ref 52.

b DoT factors calculated by dividing the concentration of the compound by its odor threshold.n.a: these odorants could not be quantitated during quantitation.

In the scrambled chicken egg, 14 of the 17 quantitated odorants exhibited DoT factors ≥ 1, highlighting their significant impact on the overall aroma. Among them, (*E,Z*)-2,6-nonadienal displayed the highest DoT factor of 1274, followed by 3-methylbutanal (439) and 3-(methylsulfanyl)­propanal (418), both of which are key Strecker aldehydes contributing with malty and potato-like notes. DoT factors of 2,3-butanedione (230) and H_2_S (97) were also prominent contributors, providing buttery and sulfury notes characteristic of scrambled egg aroma. Additionally, compounds such as 2-methylbutanal (DoT factor of 75) and 1-octen-3-one (DoT factor of 63) added malty and mushroom-like nuances to the profile. Other contributors included hexanal (DoT factor of 47) and (*E*)-2-nonenal (DoT factor of 20) imparted grassy, green, fatty, and cardboard-like aroma, respectively.

In contrast, the scrambled vegan egg exhibited a different aroma profile, with 12 of the 13 quantitated odorants showing DoT factors ≥ 1, most of them having lower DoT factors as compared to the scrambled chicken egg. Notably, 3-methylbutanal had the highest DoT factor of 94, indicating its dominant role in shaping the vegan egg’s malty aroma. 3-Methylbutanoic acid (DoT factor of 4) and 2-methylbutanoic acid (DoT factor of 3), while present in higher concentrations than in chicken eggs, showed moderate DoT factors due to their high odor threshold. However, their presence still underscores their significant contribution to the cheesy and fatty notes characteristic of the vegan egg. Lipid oxidation products, such as hexanal (DoT factor of 51) and *trans*-4,5-epoxy-(*E*)-2-decenal (DoT factor of 88), were significant contributors to the vegan egg’s green and metallic notes, comparable to their contributions in chicken eggs. Interestingly, 1-octen-3-one (DoT factor of 79) showed a high DoT factor in the vegan egg, indicating its role in earthy and mushroom-like notes. Sulfur-containing compounds also played a role in the vegan egg aroma, with H_2_S (DoT factor of 50) and methanethiol (DoT factor of 30), although they were generally less pronounced compared to the chicken egg.

Several compounds in both matrices exhibited DoT factors < 1, suggesting minimal contributions to the overall aroma. For example, 4-hydroxy-2,5-dimethyl-3­(2*H*)-furanone, 3-methylbutanoic acid, and 2-methylbutanoic acid had a low DoT factor in the chicken egg, reflecting its limited impact despite its presence. In the vegan egg, 2-methylbutanoic acid and acetic acid also exhibited DoT factors < 1, indicating a limited sensory impact despite their detection.

### Aroma Reconstitution Studies and Omission Experiments

The calculation of dose over threshold factors (DoT factors) offers valuable insights into the aroma potency of individual compounds within a given matrix. However, studies have shown that the aroma of a mixture is often dominated by specific odorants, while others with DoT factors clearly >1 may be completely suppressed.^50^ To identify the crucial compounds responsible for the overall aroma perception, aroma reconstitution and omission experiments were essential, as an integral part of the sensomics approach, using a matrix closely resembling that of cooked chicken eggs. Three aroma reconstitution models were prepared and submitted for evaluation as described above, with their aroma profile presented in Figure 2 and the degree of resemblance to scrambled eggs shown in Figure 3. As outlined in Table 4, the first reconstitution model (RM1) included all 12 odorants with DoT factors of ≥ 1. The second model (RM2) focused on the seven odorants with the highest DoT factors (DoTs ranging from 63 to 1274), excluding five compounds with lower DoT factors (ranging from 4 to 47). This strategy aimed to assess whether these lower impact compounds contributed meaningfully to the overfall aroma or were masked by higher impact odorants. The third model (RM3) excluded H_2_S, a compound well known for its critical contribution to the eggy and sulfury aroma of chicken eggs.

**Table 4:** Aroma Reconstitution Models of the Scrambled Chicken Egg^a^

	reconstitution models		
odorants	RM 1	RM 2	RM 3
(*E*,*Z*)-2,6-nonadienal	x	x	x
3-methylbutanal	x	x	x
3-(methylsulfanyl)propanal (methional)	x	x	x
2,3-butanedione	x	x	x
H_2_S	x	x	
2-methylbutanal	x	x	x
1-octen-3-one	x	x	x
hexanal	x		
2-ethyl-3,5-dimethylpyrazine	x		
(*E*)-2-nonenal	x		
4-methylphenol (*p*-cresol)	x		
octanal	x		

a x: compounds that were included in the reconstitution model.

The first aroma model (RM 1) containing all quantitated compounds with DoT factor ≥ 1 had a relatively rounded profile with only nutty and metallic attributes being the least intensive (Figure 2I). The result showed that most participants (67%) can associate RM 1 with egg-based dishes (Figure 3A) and 63% of participants resembled scrambled egg aroma (Figure 3B), confirming the successful simulation of RM 1. The omission of hexanal, 2-ethyl-3,5-dimethylpyrazine, (*E*)-2-nonenal, octanal, and 4-methylphenol in RM 2 at first reduced the overall aroma character of scrambled eggs (Figure 3B), shifting it toward a boiled egg sensation (Figure 3A). This phenomenon can be explained in the consensus profiling result, in which RM 2 was observed with a reduction of most attributes, whereas the sulfury and seasoning-like odor remained the same. However, to our surprise, the omission model RM 3 with only six odorants was rated to have more resemblance to the scrambled egg aroma than RM 1, with a 75% response in total (Figure 3B). Further removal of H_2_S in RM 3 clearly had a significant effect on the aroma profile as compared to RM 1, noticeably reducing sulfury, malty, seasoning-like, and metallic-like flavor intensity while increasing nutty aroma (Figure 2). This simpler combination of odorants might have resulted in a more balanced odor profile of the scrambled chicken egg. The interaction of certain odorants that produce a novel aroma impression that is not perceivable in the individual components has been reported before. The combination of methional and (*Z*)-1,5-octadien-3-one in a 100:1 ratio has been shown to create a fishy odor of dry spinach.^51^ Similarly, the distinctive aroma of fresh walnut can be successfully recreated using only sotolon and (*E,E,Z*)-2,4,6-nonatrienal.^21^ Hence, findings in this study prompted the hypothesis that a comparable interaction might contribute to the characteristic aroma of scrambled chicken eggs. Nevertheless, the aroma reconstitution and omission experiments in this study have successfully discovered the six critical odorants of scrambled chicken eggs, notably excluding H_2_S. The latter is likely to play a more pronounced role in boiled eggs, while when scrambled eggs are presented and consumed, H_2_S was markedly lost by evaporation or interactions.

In summary, our study revealed a significant gap in odor-active compounds between chicken scrambled eggs and their plant-based counterparts. The most important drivers for the overall aroma of scrambled chicken eggs were characterized, and it was demonstrated that only six compounds were sufficient to successfully recreate the aroma profile. This study constitutes an important basis for the flavor optimization of plant-based egg alternatives, which can be achieved in the next steps by using selected plant-based raw materials with respective flavor precursors that might generate a desired aroma upon heating. Future formulation strategies can thus be designed to target the generation of these key odorants found in scrambled chicken eggs, enabling the development of plant-based egg products with a sensory profile that more closely mimics that of conventional eggs.

## Supplementary Material



## Notes

Stable isotopically substituted odorants and parameters used in the quantitation of odor-active compounds; parameters used in the quantitation of acetic acid, H_2_S, and methanethiol; and list of reference odorants used in consensus profiling (PDF)

## Abbreviations used

AEDA: aroma extract dilution analysis
CI: chemical ionization
DoT: dose over threshold
EI: electron ionization
FD: flavor dilution
GC–MS: gas chromatography–mass spectrometry
GC–O: gas chromatography–olfactometry
GC–GC–MS: two-dimensional gas chromatography–mass spectrometry
OT: odor threshold
RI: retention index
SAFE: solvent-assisted flavor evaporation.
