# Supplementary material for: Decoding the Aroma Gap: Sensomics-Based Characterization of Key Odorants in the Plant-Based Egg and Chicken Egg
Source: J Agric Food Chem. 2025 May 22;73(22):13832–42. doi: 10.1021/acs.jafc.5c04304 (PMC12147153; doi:10.1021/acs.jafc.5c04304)
Supplement: Supplementary file 1 [file jf5c04304_si_001.pdf]

Decoding the Aroma Gap: Sensomics based Characterization of Key Odorants in Plant-Based Egg and Chicken Egg

Thi Khanh Linh Tran, Elodie Gillich, Amandine André, Marie-Louise Cezanne, Peter Gläser, Imre Blank, Irene Chetschik*

Zurich University of Applied Sciences (ZHAW), Life Sciences and Facility Management, 8820 Wädenswil, Switzerland

---

*Corresponding authors:

Irene Chetschik

Phone: +41 58 934 5651

Fax: +41 58 934 5001

E-mail: irene.chetschik@zhaw.ch

Supporting Information **Table S1. Stable Isotopically Substituted Odorants and Parameters used in the Quantitation of Odor-Active Compounds**

target compound	isotopically substituted odorant as internal standard	quantifier ions (*m/z*)		calibration line[a]	R^2	quantitation system
		analyte	standard			
2,3-butanedione	[$^{13}C_4$]-2,3-butanedione	87.09	91.09	y = 1.6042 x + 0.6198	0.9996	II (PCI)
4-hydroxy-2,5-dimethyl-3(2*H*)-furanone	[$^{13}C_2$]-4-hydroxy-2,5-dimethyl-3(2*H*)-furanone	128	130	y = 1.0392 x + 0.0035	0.9999	I
4-methylphenol	[$^2H_2$]-4-methoxyphenol	107	108	y = 0.7428 x + 0.2427	0.9999	I
(*E,Z*)-2,6-nonadienal	[$^2H_5$]-(*E,Z*)-2,4-nonadienal	120	125	y = 1.6042 x + 0.6198	0.9938	II (EI)
octanal	[$^2H_4$]-octanal	110	114	y = 0.5816 x + 0.0983	0.9972	II (EI)
2,3-diethyl-5-methylpyrazine	[$^2H_7$]-2,3-diethyl-5-methylpyrazine	150	155 & 157	y = 1.3234 x + 0.155	0.9999	I
2-methylbutanoic acid	[$^2H_9$]-3-methylbutanoic acid	74	63	y=0.9073x−0.0149	0.9957	I
3-methylbutanoic acid	[$^2H_9$]-3-methylbutanoic acid	60	63	y=0.9073x−0.0149	0.9998	I
(*E*)-2-nonenal	[$^2H_2$]-(*E*)-2-nonenal	111	113	y = 0.4835 x + 0.1936	0.9885	II (EI)
1-octen-3-one	[$^2H_3$]-1-octen-3-one	70	73	y = 0.0096 x - 0.0003	0.9979	II (PCI)
2-ethyl-3,5-dimethylpyrazine	[$^2H_5$]-2-ethyl-3,6-dimethylpyrazine	135	139 & 141	y = 1.214 x - 0.0515	0.9992	I
2-ethyl-3,6-dimethylpyrazine	[$^2H_5$]-2-ethyl-3,6-dimethylpyrazine	135	139 &141	y = 1.7521 x - 0.0436	0.9972	I
3-hydroxy-4,5-dimethyl-2(5*H*)-furanone	[$^{13}C_2$]- 3-hydroxy-4,5-dimethyl-2(5*H*)-furanone	128	130	y = 0.9564 x + 0.0843	0.9991	I
hexanal	[$^2H_5$]-hexanal	72	77	y = 0.7061 x + 0.2859	0.9994	I
3-(methylsulfanyl)propanal	[$^2H_3$]-3-(methylsulfanyl)propanal	104	107	y = 1.2494 x + 0.0012	0.9992	II (EI)
2-methylbutanal	[$^2H_3$]-2-methylbutanal	57	60 & 61	y = 0.9857 x + 0.3236	0.9977	II (EI)
3-methylbutanal	[$^2H_2$]-3-methylbutanal	71	73	y = 0.2171 x + 0.1044	0.9964	II (EI)
trans-4,5-epoxy-2-(*E*)-decenal	[$^2H_2$]-*trans*-4,5-epoxy-2-(*E*)-decenal	169.12	171.12	y = 0.449 x + 0.2449	0.9926	II (PCI)
2-acetyl-1-pyrroline	[$^{13}C_5$]-2-acetyl-1-pyrroline	111	116	y = 1.08 x - 0.0185	1	II (PCI)

[a]y = peak area standard / peak area analyte; x = amount standard (µg) / amount analyte (µg)

I = GC-MS; II = GC-GC-MS; EI = electrical ionization mode; PCI = positive chemical ionization mode.

Table S2. Parameters used in the Quantitation of Acetic Acid, Hydrogen Sulfide and Methanethiol

target compound	internal standard	calibration line	R^2
acetic acid	propionic acid	$y = 0.0015x – 0.0656$	0.9929
hydrogen sulfide	-	$y = 2799.7x^2 - 286.16x + 823.94$	1.0000
methanethiol	-	$y = 1621464x - 1285$	0.9933

Table S3. List of reference odorants used in Consensus profiling.

no.	sensory attributes	odorants/materials
1	mushroom-like	1-octen-3-one
2	cooked potato-like	methional (3-(methylsulfanyl)propanal)
3	fatty	(*E*)-2-nonenal
4	malty	3-methylbutanal
5	earthy	2-ethyl-3,5-dimethylpyrazine
6	caramel-like	4-hydroxy-2,5-dimethyl-3(2*H*)-furanone
7	seasoning-like	sotolon (3-hydroxy-4,5-dimethyl-2(5*H*)-furanone)
8	metallic	*trans*-4,5-epoxy-(*E*)-2-decenal
9	nutty	2-acetyl-2-thiazoline
10	sulfury	Kala Namak salt

Table S4. Nutrient composition of chicken eggs and plant – based egg analogs

nutrients	content (*per 100g*)	
	chicken egg	vegan egg
energy (kcal)	140	150
total fat (g)	9.8	12
saturated fat (g)	2.6	1.1
trans fat (g)	0.0	0.0
polysaturated fat (g)	1.7	3.8
monounsaturated fat (g)	3.7	7.0
cholesterol (g)	0.350	0.0
sodium (g)	0.4	1.1
total carbohydrate (g)	0.3	< 0.5
dietary fiber (g)	0.0	0.0
sugar (g)	0.3	< 0.5
protein (g)	12.6	10
